# Supplementary material for: The causal relationship between the human gut microbiota and pyogenic arthritis: a Mendelian randomization study
Source: Front Cell Infect Microbiol. 2024 Nov 26;14:1452480. doi: 10.3389/fcimb.2024.1452480 (PMC11629706; doi:10.3389/fcimb.2024.1452480)
Supplement: Supplementary file 1 [file DataSheet1.zip › mendelian test/ebi-a-GCST90027501.csv_forest.pdf]

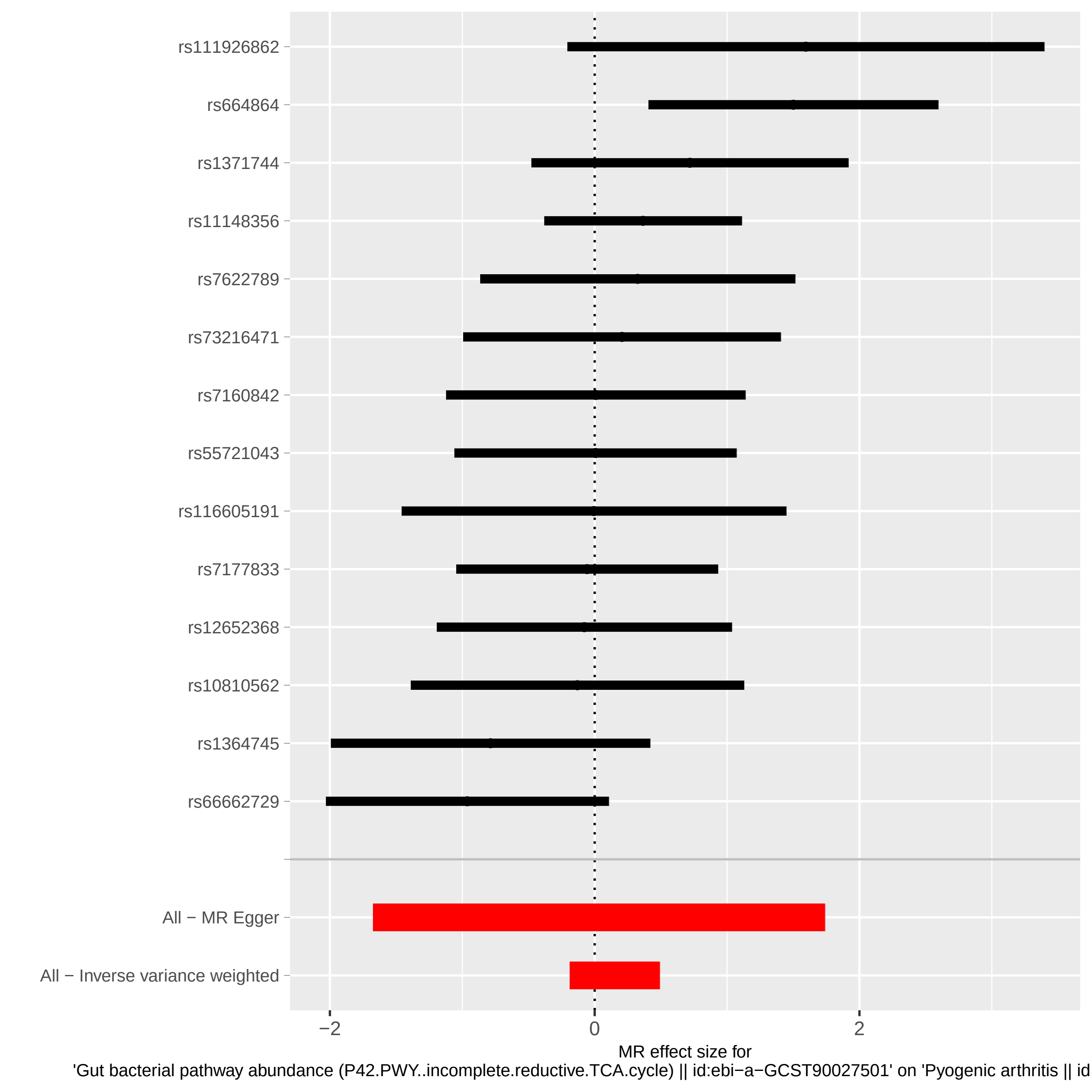
rs111926862
rs664864
rs1371744
rs11148356
rs7622789
rs73216471
rs7160842
rs55721043
rs116605191
rs7177833
rs12652368
rs10810562
rs1364745
rs66662729
All − MR Egger
All − Inverse variance weighted
−2
0
2
MR effect size for
'Gut bacterial pathway abundance (P42.PWY..incomplete.reductive.TCA.cycle) || id:ebi−a−GCST90027501' on 'Pyogenic arthritis || id
